# EHMTI-0219. Quantitative histological examinations of inter- and intraindividual differences in the dural vasculature of the mouse

**DOI:** 10.1186/1129-2377-15-S1-F19

**Published:** 2014-09-18

**Authors:** B Mecheri, F Paris, H Lübbert

**Affiliations:** 1 Department of Animal Physiology, Faculty of Biology and Biotechnology, Bochum, Germany

## Introduction

To examine both migraine and pulmonary hypertension (PH) we developed a hypoxic mouse model. Under hypoxic conditions (four weeks at 10% O2) mice develop typical symptoms of PH, characterized by an increased muscularization of arterial blood vessels in the lung. The vessel remodeling can be prevented by the chronic application of 5-HT2B receptor antagonists. We found that hypoxic mice also develop increased sensitivity towards the 5-HT2 receptor agonist meta-Chlorophenylpiperazine (mCPP). In contrast to the control group (normoxic mice kept at 20% O2) at low doses of 1 µg / kg body weight, mCPP triggers plasma protein extravasation (PPE) in the dura mater in hypoxic mice. Dural PPE is a quantifiable indicator of migraine-like events in animal models.

## Aims

The sensitization towards mCPP may coincide with structural remodeling processes in arterial blood vessels of the dura mater, possibly exhibiting similarities to vascular alterations in the lung. A prerequisite in the investigation of vascular alterations in the dura mater is a thorough understanding of the normal organization of dural vasculature in mice and of the extent of its normal biological variance.

## Methods

Histological examinations of the length of dural vessels were performed to quantify and statistically analyze inter- and intraindividual differences.

## Results

Interindividual differences: The arteriole total lengths are 15% far from the arithmetic mean.

Intraindividual differences: The difference in the length of arterioles represents 12% of the total length of the arterioles.

## Conclusion

On the basis of these results examinations of dural vessel of hypoxic mice can be investigated.

No conflict of interest.

